# New start of surgical residents training: the first survey of program directors in Korea

**DOI:** 10.1186/s12909-019-1646-3

**Published:** 2019-06-13

**Authors:** Sung Geun Kim

**Affiliations:** Division of Gastrointestinal Surgery, Department of General Surgery, Yeouido St. Mary’s Hospital, College of Medicine, The Catholic University of Korea, 10, 63-ro, Yeongdeungpo-gu, Seoul, 07345 Republic of Korea

**Keywords:** Surgical resident training, Program director, Survey, Feedback

## Abstract

**Background:**

The introduction of the 80-h shift restriction for surgical residents in Korea necessitated many changes to their training systems. The purpose of this study was to conduct a survey among program directors, determine the conditions necessary for them to fulfill their responsibilities, and investigate whether there was a difference between tertiary hospitals and general hospitals in the surgical training environment.

**Methods:**

Questionnaires were distributed to program directors nationwide to investigate their status as well as the status, conditions, evaluation methods, and feedback methods of surgical residency training programs. Descriptive statistics and the chi-square test were used for statistical analysis.

**Results:**

The response rate was 55% (55/100). These 55 institutes train 71% of all residents and 83.6% of these institutes run surgical skill training programs. A laparoscopic training box was available at 30 (55%) institutes, laparoscopic simulator at 30 (33%), and robotic simulator at only 12 (22%). Internal assessment of residents was conducted at 24 (43.6%) institutes. Regular interviews were conducted with residents at 21 (38.2%) institutes. Regular questionnaires about the training program were conducted among residents at 16 (29.1%) institutes and among training directors at 8 (14.5%). Lastly, 45 (81.8%) program directors reported that at least 30% of their working time was dedicated to residency training.

**Conclusions:**

This is the first study to elucidate current surgical residency training in Korea: Feedback systems for residency assessment and training programs are still lacking, and program directors need to dedicate at least 30% of their time to effectively fulfill their role in residency training.

## Background

In Korea, there is almost no information about whether resident training programs are being properly conducted at training institutes; whether there are appropriate regulations regarding the role, capabilities, authority, and duties of training directors; whether there are any assessments to verify that residents are completing these programs successfully; or whether the training directors are being assessed.

Until recently, surgical residents were trained by the master-apprentice doctrine and there were no rules regarding work hour limits for residents in Korea. However, since the introduction of the 80 working-hour limitation regulation in 2017, the previous training system is no longer effective [1]. In the past, it was common for residents to learn several skills on the spot while working in the operating room during the day and to work in the ward in the evening. However, after reaching their working hour limit, residents should not be in the hospital; this means that most of the ward work was done first during the day shift, which then reduced the time to meet the teaching surgeons in the operating room. Therefore, we need to teach medical knowledge and surgical skills via a systemized curriculum. In addition, as the number of night shift duties decreases, experiencing emergency situations during the night will also reduce.

The Korean Surgical Society acquired more responsibility in having to define competency and set training goals for surgical residents, and individual training institutes were required to have structured training programs that could provide surgical residents with these competencies. Therefore, it became necessary to introduce training directors or program directors who could manage the training programs and monitor and evaluate their suitability. Previously, the director (or dean) of each hospital was responsible for training, and a training director would typically be designated; however, because the training environment has changed drastically including advanced technology and techniques such as laparoscopy or robotic surgeries, there is now a greater need for a high level of expertise in training, for independent program directors to take responsibility for specialized work, and for education specialists to create and evaluate educational programs. Accordingly, in February 2018, the Korean Surgical Society requested that all training hospitals designate a program director and confirm that this has been done.

The introduction of the program director system heralds a new era of residency training. Since this is still in the initial phase, a process of trial-and-error is expected, and there is still much work to be done. The aims of this study were to conduct the first survey of designated program directors to ascertain the extent to which training institutes are prepared for surgical resident training, to investigate the conditions for the program directors to properly fulfill their responsibilities in the future, and to investigate whether there was a difference between the tertiary hospitals and the general hospitals in the surgical training environment.

## Methods

There are 102 surgical training hospitals in Korea as of 2018, and as of May 2018, two institutes do not have a designated program director, meaning that there are currently 100 designated program directors. Our survey was conducted among these 100 designated program directors.

After the questionnaire was first compiled, it went through two rounds of review and revisions with the Training Committee of the Department of Surgery at the Catholic University of Korea’s College of Medicine. The questionnaire consisted of 20 questions, including single-answer and multiple-choice questions. The questions inquired about the program director’s experience, specialties, the training program, training environments, assessment and feedback systems, as well as a question about the requirements for settlement of the program director system. The program director was educated during the Korean Surgical Society Congress in May 2018, where they were provided with a questionnaire.

This study was performed according to the Helsinki Declaration, and the Institutional Review Board of the Catholic University of Korea, College of Medicine approved the study protocol (approval number: SC17RES10080). Informed consents were obtained from all participants.

### Statistical analysis

Descriptive statistics were used to analyze program directors’ characteristics. The content of the residency training programs was compared between two groups: group A consisted of tertiary hospitals with at least 12 residents, and group B consisted of general hospitals with 4–11 residents. The chi-square test was used, with *P*-values < 0.05 considered as statistically significant. All statistical analyses were performed using SPSS, version 18.0 (SPSS, Inc., Chicago, IL, USA).

## Results

### Program directors

Responses were received from 55 program directors, representing 55% of program directors from all 100 institutes. Four hundred twelve residents being trained at institutes with program directors responded, representing 71% of the 580 surgical residents as of March 2018. Twenty-five institutes (45%) had 12–48 residents enrolled in the program per year, while 30 (55%) had 4–11 per year. The mean number of residents per year among all hospitals was 4 persons, with a wide range from 1 to 12 persons (Table 1). In terms of program director experience, the median time since receiving board certification for surgery was 13 years (range 5–32 years), and the specialties were, in descending order, breast and endocrine, hepato-biliary-pancreatic, colorectal, gastrointestinal, vascular/transplantation, traumatic/intensive care, and pediatric surgeon (Table 1).

**Table 1:** Characteristics of program directors of surgical residency training programs

Demographics	N	(%)
Duration of professional career (years)		
> 20 years	2	(3.6%)
15–20 years	13	(23.6%)
10–15 years	20	(36.4%)
< 10 years	20	(36.4%)
Subspecialty		
BE	14	(25.5%)
HBP	13	(23.6%)
CR	12	(21.8%)
GI	7	(12.7%)
VA	2	(3.6%)
IT	2	(3.6%)
P	1	(1.8%)
No response	4	(7.3%)
Enrolled residents		
12–48	25	(45.5%)
5–11	17	(30.9%)
4	13	(23.6%)
Sufficient time for resident training		
yes	4	(7.3%)
no	51	(92.7%)
Guaranteed dedicated training time		(0.0%)
20%	10	(18.2%)
30%	16	(29.1%)
40%	11	(20.0%)
> 50%	18	(32.7%)

*BE* breast and endocrine surgery, *HBP* hepato-biliary-pancreatic surgeon, *CR* colorectal surgeon, *GI* gastrointestinal surgeon, *VA* vascular/transplant surgeon, *IT* traumatic/intensive care surgeon, *P* pediatric surgeon

### Training program

Of the participating institutes, 73% (40/55) offered residents a written training program, while 46 institutes (83.6%) offered residents surgical skill training. Regular staff lectures were provided at 46 institutes (83.6%) and not provided at 9 institutes (Table 2). There were no statistically significant differences between groups A and B in the training programs.

**Table 2:** Characteristics of the resident training program

	Group A	Group B	TotalN (%)	*p*-value
	(no. of residents ≥12)	(no. of residents < 12)		
Documented training program				0.472
yes	17	23	40 (72.7%)	
no	8	7	15 (27.3%)	
Surgical skill training program				0.947
yes	21	25	46 (83.6%)	
no	4	5	9 (16.4%)	
Regular staff lecture				0.947
yes	21	25	46 (83.6%)	
no	4	5	9 (16.4%)	

*No* number

### Surgical skill training tools

In response to questions about tools for surgical skills training, 45 institutes (82%) reported possession of a basic suture kit, 30 (55%) had a laparoscopic training box, 18 (33%) had a laparoscopic surgery simulator, and 12 (22%) had a robotic surgery training simulator. An animal laboratory training center was available for use at 12 institutes (22%). Fifty-four institutes (98%) offered a surgical skill workshop for surgical residents based on these tools and facilities. These workshops were financed by the hospital or department of surgery itself in 50% of cases and supported by other sources for the remaining 50%. Animal laboratories were reported more often in group A than in group B, but the difference was not statistically significant (Table 3).

**Table 3:** Availability of surgical skill training tools

	Group A	Group B	Total N (%)	*p*-value
Basic suture kit				0.750
yes	20	25	45 (81.8%)	
no	5	5	10 (18.2%)	
Laparoscopic training box				0.843
yes	14	16	30 (54.5%)	
no	11	14	25 (45.5%)	
Robot simulator				0.721
yes	6	6	12 (21.8%)	
no	19	24	43 (78.2%)	
Animal laboratory				0.095
yes	8	4	12 (21.8%)	
no	17	26	43 (86.7%)	
Laparoscopic simulator				0.637
yes	9	9	18 (32.7%)	
no	16	21	37 (67.3%)	

### Assessment tool

For resident assessment, 24 institutes (43.6%) reported using an in-house tool. Among these 24 institutes, 6 assessed residents monthly, 6 every semester, 12 biannually, and 1 institute used a different assessment cycle. Resident assessment forms were kept on-site at 17 institutes (31%). An independent test to assess the medical knowledge of residents was used at 18 institutes (33%). There were no differences between the two groups in responses to these questions (Table 4).

**Table 4:** Evaluation methods of the surgical residency training programs

	Group A	Group B	Total N (%)	*p*-value
Independent evaluation tool				0.414
yes	9	15	24 (43.6%)	
no	16	15	31 (56.4%)	
Evaluation cycle				0.247
monthly	1	4	5 (9.1%)	
every 3 months	1	5	6 (10.9%)	
every 6 months	7	5	12 (21.8%)	
other		1	1 (1.8%)	
Medical knowledge test				0.916
yes	8	10	18 (32.7%)	
no	17	20	37 (66.7%)	

### Feedback

Twenty-one institutes (38%) conducted regular interviews with the residents, and 14 (25%) kept a record of these interviews. Questionnaires about training were regularly administered to residents at 16 institutes (29%), while 8 institutes (15%) regularly administered questionnaires about residency training to the training directors. Ten institutes (18%) provided education in the training program for training directors based on the results of the questionnaires. This education for training directors was provided more commonly in group B than in group A, but this difference was not statistically significant (Table 5).

**Table 5:** Residency training program feedback

	Group A	Group B	Total N (%)	*p*-value
Regular interview with residents				0.156
yes	7	14	21 (38.2%)	
no	18	16	34 (61.8%)	
Retained interview records				0.397
yes	5	9	14 (25.5%)	
no	20	21	41 (74.5%)	
Surveys from residents				0.175
yes	5	11	16 (29.1%)	
no	20	19	39 (70.9%)	
Surveys from trainers				0.625
yes	3	5	8 (14.5%)	
no	22	25	47 (85.5%)	
Tutor workshop				0.074
yes	2	8	10 (18.2%)	
no	23	22	45 (81.8%)	

### Dedicated time

Among program directors, only 4 persons (7%) reported having sufficient time for residency training, and the other 51 (93%) reported a lack of time. When asked how much of their own working time they had to commit to residency training, more than half of the program directors (81.8%) reported that it was “30% or more” (Table 1).

## Discussion

The qualifications for board certification in Korea are defined in the President’s decree, “Regulations Relating to the Training and Accreditation of Board Certified Physicians”; whereas the minimum definition and content for residency training are described in a notice by the Ministry of Health and Welfare titled, “Training Curriculum for Resident Physicians By Year” [1, 2]. The training curriculum includes information regarding the scope of patients treated, the minimum number of surgeries, knowledge, surgical skill, in-training examination, conference attendance, and thesis submission. However, specific details are missing; for example, in the stipulations regarding the minimum number of surgeries, the information is ambiguous. And the minimum operative experience is significantly lower than the minimum operative experience in the US, at 400 surgeries over a span of 4 years. [2]. Moreover, the regulations do not refer to the education philosophy or goals, but are simply a list of conditions for acknowledgment of qualification [3].

In Korea, there is no organization responsible for preparing standardized criteria for residency training programs and surgical training institutes, or for assessing and certifying whether appropriate programs are being developed and implemented. As a result, residency training has been left to the autonomous training programs at each institute, and considerable differences have emerged between institutes. As part of efforts to standardize residency training, the Korean Surgical Society developed skills and academic training programs for residents since 2006 and made efforts to improve the quality and quantity of these programs. However, these cannot replace the training programs at individual training institutes.

In the United States, approval of residency training and other graduate medical education is conducted by the Accreditation Council for Graduate Medical Education (ACGME) [4]. Accreditation of board-certified physicians is recommended by the American Board of Medical Specialties (ABMS). Each training institute must have their training programs approved, and the training goals and content must adhere to the basic frameworks provided by the ACGME and ABMS. Based on this, each training institute needs to have a structured training program. The situation is the same in other advanced countries, such as the United Kingdom, Denmark, and Germany [5]. The essential content that needs to be included pertains to knowledge, skills, attitude, professionalism, and competency, and criteria need to be set for each of these elements [6]. With these training programs being recommended, it became necessary to have someone responsible for the residents and training directors, and this person is the program director or professional trainer [6]. Dedicated time is essential to fulfill this role. The ACGME in the US states that 30% of surgical residency program directors’ time must be dedicated to education, and this may require their institutes to release them from clinical and other activities during their work schedule [7].

Specific legislation for residents and physicians in Korea restricts working hours to no more than 80 h per week [8]. Before this legislation, surgical residents had no working time restriction. This simple reduction in working hours means that the entire training paradigm needs to be changed. The old apprenticeship system is no longer tenable, and it has become necessary to implement skill-based training and evaluation through structured training programs, in which objectives are set and achievement of these objectives is assessed.

The Korean Surgical Society named the training directors who fulfill the above roles as “program directors”. From June 2017, they formed a task force team that created regulations for program directors, and in February 2018, they required all 102 training institutes in the country to designate a program director. In March 2018, the first Program Director Workshop was held, which was the first attempt to formalize a system for program directors in Korea. Indeed, since this is only the beginning, regulations are still incomplete, and because of the volume of work and lack of a support system, a significant burden is placed on those who have been designated as program directors. Nevertheless, if training programs are not well-prepared and their performance is not evaluated, these organizations can no longer fulfill their responsibilities as training institutes. If these conditions are not met, The Korean Surgical Society will suspend the institute’s certification as a training hospital. Program directors at each institute must perform the important task of perfecting training programs and preparing assessment systems.

In terms of experience, the program directors had, on average, obtained board certification 12 years ago, and there were 11 program directors who received board certification at least 15 years ago, representing 20% of the respondents. In comparison to a survey in the United States, in which 56.2% of respondents were for at least 50 years old, this shows that training directors are generally younger in Korea [9].

The availability of a written residency training program indicates that it is possible to start a structured training program. However, the fact that 27% of institutes were still unable to provide residents with a structured training program is thought to reflect the current period of transition, moving from apprenticeship-based education to systematic education for residents.

The increase in laparoscopic surgery has brought significant environmental changes to the acquisition of surgical skills [10]. It has become more difficult for the surgeon to assess the assistant’s surgical ability in the operative field, and this has led to fewer opportunities for residents to perform surgery. As a result, various practice instruments and methods have become necessary for learning surgical skills. In our survey, the percentages of institutes with a laparoscopic training box, laparoscopic surgery simulator, and robotic surgery training simulator were similar to those in a 2006 survey in the United States, where only 55% of training hospitals were equipped with skills laboratories. However, after the ACGME stipulated that training hospitals must have a simulation and skills laboratory for surgical training, a survey in 2013 found that 99% of all training hospitals were equipped with a simulation laboratory, and 63% had an organized simulation curriculum [11]. This highlights the importance of increasing investment and establishing regulations regarding skills training at training institutes in Korea.

Korea’s Ministry of Health and Welfare’s Training Environment Assessment Committee is responsible for assessing the residency training environment. The regulations are consolidated annually, and recently the committee has examined whether elements of resident assessment exist and whether individual clinical departments have their assessment tool. In our survey, 45% of institutes had their own assessment tool within the surgical department. This percentage is slightly lower than that from a survey conducted in Japan in 2016, where 55% of surgical training institutes had their tool for assessing the capabilities of residents [12]. However, only 31% of training institutes stored its assessment forms. This finding demonstrates that assessment of residency training programs is still lacking, and that the future level of utilization is undecided.

One of the most essential roles of program directors is to interview the residents. These interviews need to be conducted regularly and on an individual basis. Twenty-one institutes (38%) reported having regular interviews with the residents, meaning that a large number of institutes still do not conduct regular interviews. Up until now, in surgical departments especially, the level of bonding between training directors and residents has been high in Korea. As a result, there is a traditional sense that the training directors and residents know and understand each other without the need for separate interviews, which, to some extent, leads to regular interviews being perceived as awkward or unnatural. However, given the workload of training directors and the fact that it has become difficult to encounter residents outside of working hours, the bond with residents has become much weaker when regular interviews are not scheduled. Therefore, training institutes should make efforts to conduct interviews with residents at appropriate intervals.

In order to ascertain whether residency training programs are being properly implemented, it is essential to receive feedback from the residents participating in the training and to hear the thoughts of the training directors directly involved in the training [6]. In our survey, feedback was obtained from residents at only 29% of training institutes and from training directors at only 15%. These data indicate the need to acquire the opinions of the trainers and trainees in order to develop more effective training programs.

For the first time, most individuals (93%) taking on the role of program director reported not being able to dedicate enough time to the training. This result is not surprising given that the program directors are not institutionally guaranteed dedicated time. Moreover, education does not traditionally offer any reward relative to treatment or research, so it is usually relegated on the list of priorities and left to be completed in one’s spare time. In order for program directors to perform their roles properly, most (82%) reported that they would need to be able to use at least 30% of their working time on training, demonstrating the importance of the program director’s role and that a significant amount of time needs to be dedicated to training in order to perform that role properly.

This study has a couple of limitations. First, because the results represent 55% of all surgical training institutes, it could be considered to not be representative of the whole. However, given that the institutes included in the survey are responsible for training 71% of all residents, we believe that the results are significant. Second, this study was a questionnaire-based survey so individual responses could not be verified. The accuracy of the study could be questioned because we were unable to obtain certain information, such as the level of completeness of the training programs, function and scale of the surgical skills laboratories, adherence to assessment tables for residents, and detailed interview records. Since our results are based on the sincere responses of program directors, who are responsible for training at each institute, we believe that criteria could be prepared and amended to determine quantitative differences in subsequent field surveys.

## Conclusions

This survey provides the first results examining the current state of surgical residency training in Korea. Most institutes have structured residency training programs that are being delivered to residents. However, the level of resident assessment is still insufficient. Feedback activities to improve training programs, based on information obtained through interviews and questionnaires, are still in their early stages. There was little difference between tertiary hospitals with a large number of residents and general hospitals with a small number of residents. The opinion of most respondents was that at least 30% of program directors’ working time would need to be dedicated to training. We expect that the results of this survey will provide primary data for the development of standardized, structured, surgical training programs.

## Data Availability

The datasets created and analysed in the current study are available from the corresponding author on reasonable request.
